# Supplementary material for: Identification of the Axis β-Catenin–BTK in the Dynamic Adhesion of Chronic Lymphocytic Leukemia Cells to Their Microenvironment
Source: Int J Mol Sci. 2023 Dec 18;24(24):17623. doi: 10.3390/ijms242417623 (PMC10744074; doi:10.3390/ijms242417623)
Supplement: Supplementary file 1 [file ijms-24-17623-s001.zip › ijms-2735807-supplementary.pdf]

Supplementary information of:

Identification of the Axis β-Catenin–BTK in the Dynamic Adhesion of Chronic Lymphocytic Leukemia Cells to Their Microenvironment

Imane Mihoub [1,2,†], Tareck Rharass [1,2,†], Souhaïl Ouriemmi [1,2], Antonin Oudar [1,2], Laure Aubard [1,2], Valérie Gratio [3], Gregory Lazarian [1,2,4], Jordan Ferreira [1,2], Elisabetta Dondi [1,2], Florence Cymbalista [1,2,4], Vincent Levy [5], Fanny Baran-Marszak [1,2,4], Nadine Varin-Blank [1,2,*], Dominique Ledoux [1,2], Christine Le Roy [1,2‡] and Laura Gardano [1,2,*,‡]

[1] INSERM, U978, 93000 Bobigny, France; mihoub.imane993@gmail.com (I.M.); souhail.ouriemmi@univ-paris13.fr (S.O.); antonin.oudar@inserm.fr (A.O.); laure.aubard@inserm.fr (L.A.); gregory.lazarian@aphp.fr (G.L.); jordan.ferreira@univ-paris13.fr (J.F.); elisabetta.dondi@inserm.fr (E.D.); florence.cymbalista@aphp.fr (F.C.); fanny.baran-marszak@aphp.fr (F.B.-M.); ledoux@univ-paris13.fr (D.L.); christine.le-roy@inserm.fr (C.L.R.)
[2] UFR SMBH, LabEx INFLAMEX, Université Paris 13—«Sorbonne Paris Nord», 93000 Bobigny, France
[3] INSERM U1149, Université Paris Cité, Hôpital Bichat, 75018 Paris, France; valerie.gratio@inserm.fr
[4] AP-HP Hôpital Avicenne, 93000 Bobigny, France
[5] URC, AP-HP Hôpital Avicenne, 93000 Bobigny, France; vincent.levy@aphp.fr
* Correspondence: nadine.varin@inserm.fr (N.V.-B.); laura.gardano@univ-paris13.fr (L.G.)
† These authors contributed equally to this work.
‡ These authors also contributed equally to this work.

Supplementary Figure legends

Supplementary Figure S1. A) Representative cytometry plot of adhesion dynamic analysis in co-culture of Hg-3 and HS-5 cells. The time points indicated correspond to the duration of the co-culture of Hg-3 and HS-5 with a ratio 5:1. **B)** Quantification of the ratio of Hg-3 cells and HS-5 cells after the removal of non-adherent Hg-3 cells. There is no statistical significant difference in the number of Hg-3 recovered with the HS-5 cells upon the time of co-culture indicated. The graph represents the data collected from 3 independent co-cultures.

Supplementary Figure S2. The gating strategy used in flow cytometry shows the percentage of ◎-catenin$^+$ in Hg-3 cells in adherent *versus* non-adherent cells to human stromal cells (HS- 5). The selection of Hg-3 cells is based on CD19 expression, whereas HS-5 is based on CD90 expression.

Supplementary Figure S3. A) Image stream images of cell doublets composed of Hg-3 cells labelled and HS-5 cells (CD19 negative) to show the localization of ◎-catenin in the area of

contact between the two cell types. Hg-3 cells are CD19-PE positive cells and HS-5 are CD19 negative. b-catenin was labelled with an antibody coupled to A488 fluorochrome. **B)** Protein cell extracts from HEK293T cells transfected with empty plasmid (Mock) or human BTK gene were analyzed by immunoblot and probed with an antibody against BTK or its phosphorylated form (pY223). ⊚-tubulin was used to assess equal loading. Cell extracts were loaded on two membranes and probed with anti-p-BTK (pY223) and anti-total BTK respectively. The loading controls of the two membranes are shown.

Supplementary Figure S4. Top panel. Flow cytometry gating strategy used for the detection of intracellular activated pBTK (pY223) in purified primary CLL B cells. Two patient samples (UPN 212 & 256) are shown. CLL cells were selected for expression of CD19 and CD5 and the gate for pY223 BTK positivity was placed on the FMO signal (not shown). Bottom panels represent the analysis of pBTK in co-culture of primary cells with HS-5 cells for the indicated time points. Percentages of pBTK (pY223) positive cells and pBTK MFI of the same samples are shown.

Supplementary Figure S5. A) RT-qPCR was used to detect Axin-2 transcript in Hg-3 cells transfected with ⊚-catenin S45A or an empty vector and co-cultured or not with HS-5 cells for 4 hours. **B**) RT-qPCR was used to detect Axin-2 transcript from 4 patient samples co-cultured with HS-5 cells or not (UN) for 4 hours. **C)** RT-qPCR of total mRNA extracted from Hg-3 cells transfected with an empty plasmid or a plasmid containing the mutated ⊚-catenin (S45A) and co-cultured or not with HS-5 cells for 4 hours. c-Myc is a Wnt/TCF and NF-⊚B target gene. Dll1 and Hes 1 were used as Notch and NF-⊚B target genes, respectively. *$p<0.05$, Student t-test.

Supplementary figure S1

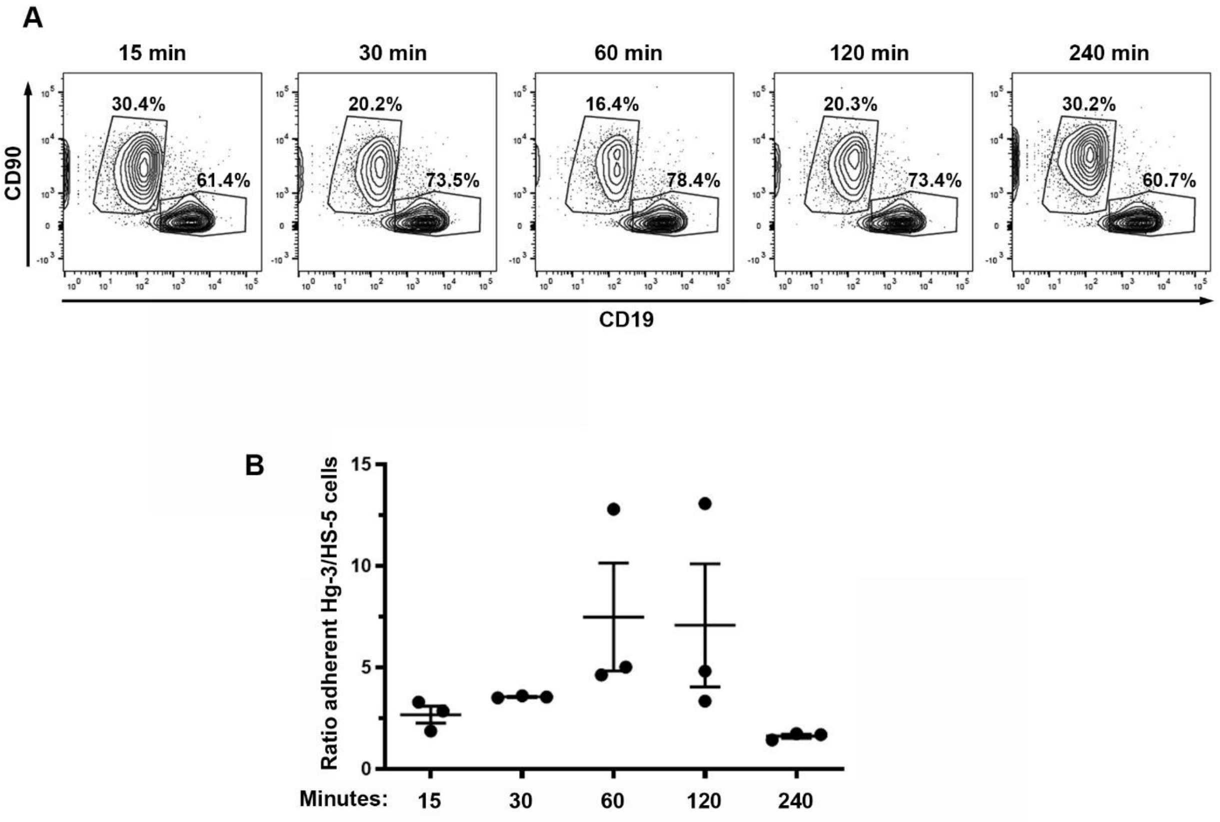

Supplementary figure S2

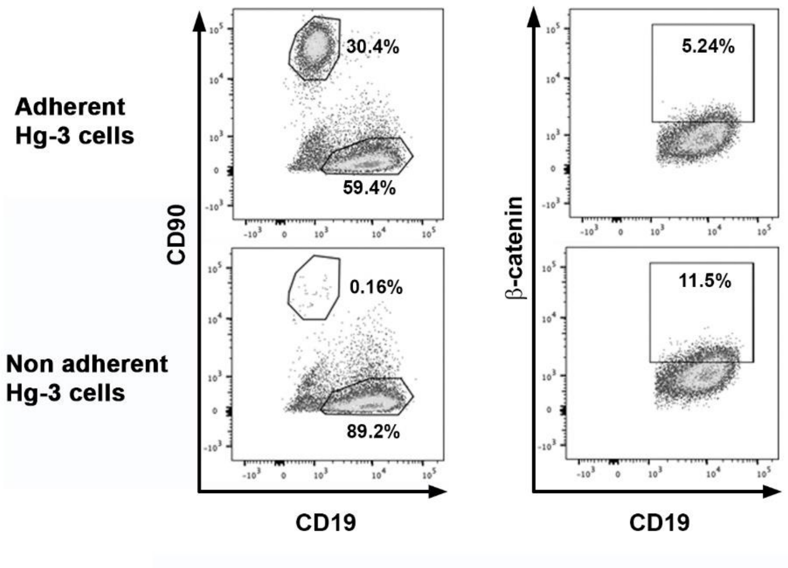

Supplementary figure S3

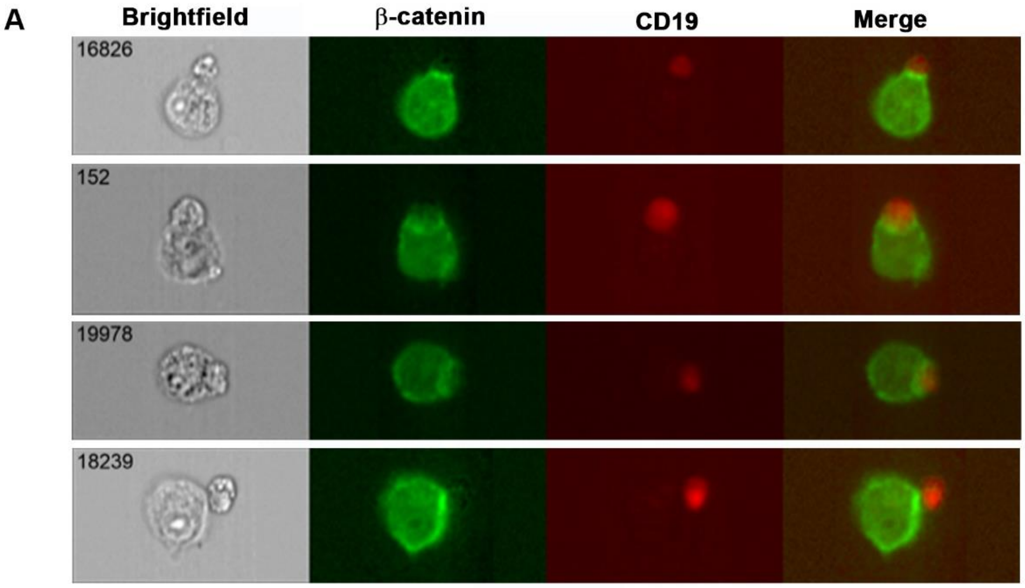

Coculture HS-5 / Hg-3

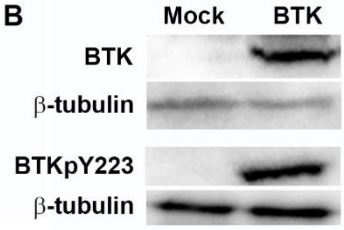

Supplementary figure S4

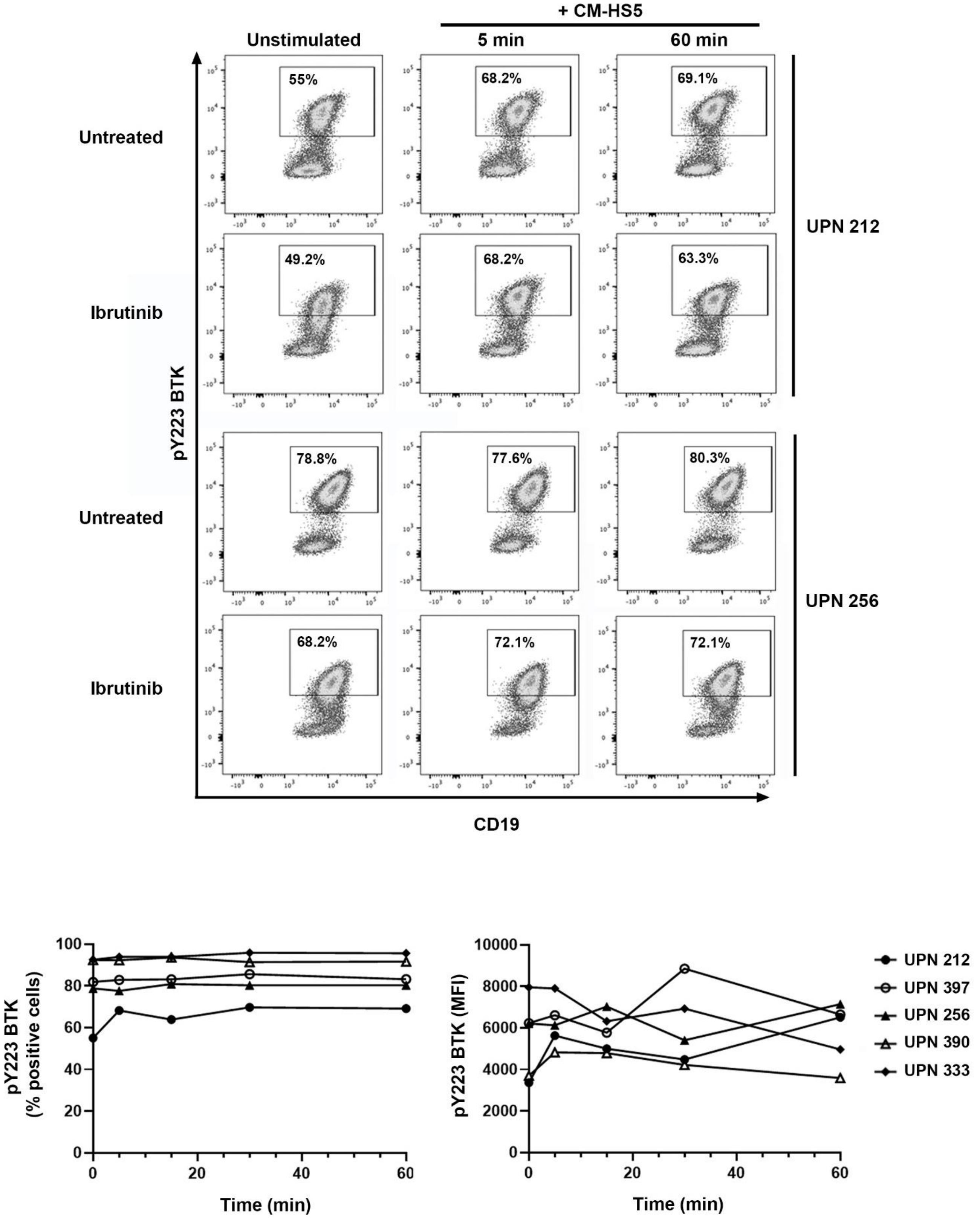

Supplementary figure S5

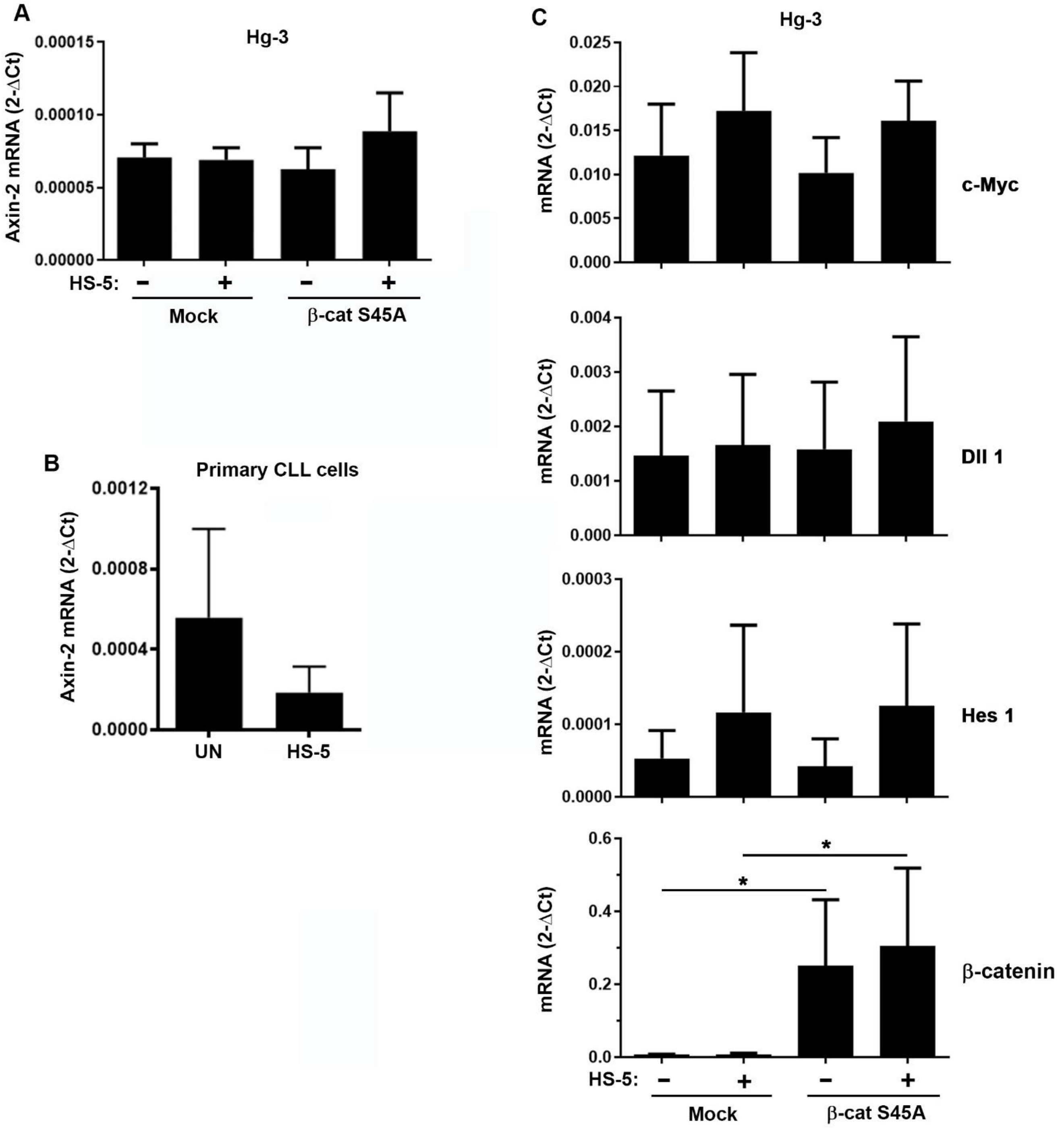

Supplementary Table S1: Characteristics of CLL patients

UPN	Gender	Age	Lymphocytosis (10^9/L)	IGHV mutational status
19	M	53	117	UM
66	F	76	193	UM
103	M	68	103	M
108	M	51	160	M
161	M	77	51	M
173	F	76	33	ND
181	M	79	113	UM
182	M	73	105	M
187	M	73	97	M
198	M	65	129	UM
203	M	89	118	M
208	M	57	51	M
212	M	65	169	M
256	M	57	97	M
263	M	68	12	M
296	M	83	340	M
285	M	77	74	M
298	M	81	207	M
299	M	72	107	M
307	F	67	38	M
319	F	82	48	UM
331	F	80	88	M
333	F	80	222	M
338	M	90	22	M
347	M	87	13	UM
358	M	65	190	UM
388	F	71	101	M
390	M	65	334	UM
397	F	66	72	UM
435	F	77	46	UM
438	M	47	94	UM
439	M	70	30	UM
441	M	50	28	UM
447	F	71	166	UM

M: Mutated ; UM: Unmutated; ND: Not determined
